# In Situ SERS Sensing by a Laser-Induced Aggregation of Silver Nanoparticles Templated on a Thermoresponsive Polymer

**DOI:** 10.3390/bios12080628

**Published:** 2022-08-11

**Authors:** Larisa V. Sigolaeva, Natalia L. Nechaeva, Anton I. Ignatov, Lyubov Y. Filatova, Timur Z. Sharifullin, Jonas Eichhorn, Felix H. Schacher, Dmitry V. Pergushov, Alexander M. Merzlikin, Ilya N. Kurochkin

**Affiliations:** 1 Department of Chemistry, M.V. Lomonosov Moscow State University, 119991 Moscow, Russia; 2 N.M. Emanuel Institute of Biochemical Physics of Russian Academy of Sciences, 119334 Moscow, Russia; 3 All-Russia Research Institute of Automatics, 127055 Moscow, Russia; 4 National Research Moscow State University of Civil Engineering, 129337 Moscow, Russia; 5 Institute of Organic Chemistry and Macromolecular Chemistry (IOMC), Friedrich-Schiller-University Jena, D-07743 Jena, Germany; 6 Jena Center for Soft Matter (JCSM), Friedrich-Schiller-University Jena, D-07743 Jena, Germany; 7 Center for Energy and Environmental Chemistry (CEEC), Friedrich-Schiller-University Jena, D-07743 Jena, Germany; 8 N.N. Semenov Federal Research Center of Chemical Physics of Russian Academy of Sciences, 119991 Moscow, Russia; 9 Institute of Theoretical and Applied Electromagnetics of Russian Academy of Sciences, 125412 Moscow, Russia

**Keywords:** thermoresponsive, silver nanoparticles, SERS, 4-mercaptophenylboronic acid, amphiphilic diblock copolymer, poly(*N,N*-dimethylaminoethyl methacrylate), laser-induced aggregation, plasmonic heating, local laser exposure

## Abstract

A stimuli-responsive (pH- and thermoresponsive) micelle-forming diblock copolymer, poly(1,2-butadiene)_290_-*block*-poly(*N*,*N*-dimethylaminoethyl methacrylate)_240_ (PB-*b*-PDMAEMA), was used as a polymer template for the in situ synthesis of silver nanoparticles (AgNPs) through Ag^+^ complexation with PDMAEMA blocks, followed by the reduction of the bound Ag^+^ with sodium borohydride. A successful synthesis of the AgNPs on a PB-*b*-PDMAEMA micellar template was confirmed by means of UV–Vis spectroscopy and transmission electron microscopy, wherein the shape and size of the AgNPs were determined. A phase transition of the polymer matrix in the AgNPs/PB-*b*-PDMAEMA metallopolymer hybrids, which results from a collapse and aggregation of PDMAEMA blocks, was manifested by changes in the transmittance of their aqueous solutions as a function of temperature. A SERS reporting probe, 4-mercaptophenylboronic acid (4-MPBA), was used to demonstrate a laser-induced enhancement of the SERS signal observed under constant laser irradiation. The local heating of the AgNPs/PB-*b*-PDMAEMA sample in the laser spot is thought to be responsible for the triggered SERS effect, which is caused by the approaching of AgNPs and the generation of “hot spots” under a thermo-induced collapse and the aggregation of the PDMAEMA blocks of the polymer matrix. The triggered SERS effect depends on the time of a laser exposure and on the concentration of 4-MPBA. Possible mechanisms of the laser-induced heating for the AgNPs/PB-*b*-PDMAEMA metallopolymer hybrids are discussed.

## 1. Introduction

Surface-enhanced Raman scattering (SERS) is an ultrasensitive vibrational spectroscopic technique, which aims to detect analytes in close proximity to a surface of plasmonic nanostructures. It is now well understood that the plasmonic coupling effect among particles induces enormous electromagnetic enhancement that allows SERS signals to be detected with very high or even single-molecule sensitivity [1,2].

Tremendous progress in fabricating nanostructured surfaces as SERS substrates, such as ordered arrays, fractal films, metal clusters, or colloidal metal nanoparticles (NPs), has taken place due to the intensive development of nanotechnology [3,4]. In addition to the ordering of micro- or nanostructures, the distance dependence of the enhancement effect has also been reported, including the effects of the distance between adjacent nanostructures and of the distance between analyte molecules and SERS substrate. The higher surface enhancement factors show a near exponential decay with the gap distance between NPs [5].

SERS-responsive assemblies of gold or silver nanoparticles (AuNPs or AgNPs) can be formed in situ, wherein the overall dimensions and interparticle spatial distances can respond and adapt to external stimuli. Importantly, the significant enhancement of SERS spectra arises from the compulsory aggregation of AuNPs or AgNPs, leading to the formation of “hot spots” in the gap between NPs. The aggregation of NPs can be achieved by increasing the ionic strength [6], ultrasound treatment [7], or application of gradient electric field [8].

Several research groups have reported on AuNPs or AgNPs associated with a thermosensitive polymer, that is, poly(*N*-isopropylacrylamide) (PNIPAAm). These new hybrid metallopolymer materials provide a controllable spatial distribution of metal NPs in the polymer matrix via temperature variation [9,10,11] as PNIPAAm undergoes a volume phase transition from a swollen hydrated state to a shrunken collapsed state when the temperature rises above 32 °C—a lower critical solution temperature (LCST) [12]. The temperature-controlled changes in the interparticle distances and the spatial distribution of the metal NPs in thermoresponsive PNIPAAm-based metallopolymer hybrid systems considerably enhanced the SERS sensing of 4-mercaptobenzoic acid [13], 1-naphthol [14,15], dyes [16,17,18], or biologically relevant molecules such as L-tyrosine or DNA [18].

An alternative to the external heating of temperature-sensitive metallopolymer hybrids would be laser-induced local heating, which would result in the spatial approaching of metal NPs, thereby enhancing the SERS signal. A phenomenon of microscopically observed phase transition was already previously described [19,20], wherein the microparticles of collapsed PNIPAAm were formed under direct heating with a focused infrared laser within 10–700 s [19] in aqueous solutions, depending on the polymer concentration, molecular weight of the polymer, temperature, or laser power. Furthermore, the localized surface plasmon resonance of single AuNPs attached to a transparent substrate can monitor the phase transition of PNIPAAm in aqueous solution upon a laser exposure [21]. Additionally, the local laser-induced plasmonic heating of AgNPs in the thermoresponsive dextran-grafted PNIPAAm copolymer/AgNPs hybrid system has been observed [22]. Worth noting, however, is that such local SERS triggering effects in temperature-sensitive metallopolymer hybrids have not been exploited for SERS sensing applications to date.

Poly(*N*,*N*-dimethylaminoethyl methacrylate) (PDMAEMA) is also classified as a thermoresponsive polymer. Its LCST is dependent on the pH of the surrounding aqueous medium [23,24], thereby rendering this polymer dual-stimuli-sensitive. Thus, the hydrophilic–hydrophobic balance of PDMAEMA can be changed by varying both the pH and temperature. Linear PDMAEMAs and PDMAEMA-based diblock copolymers have been used as efficient surface modifiers for the design of various types of electrochemical sensors, e.g., for choline [25,26,27], phenol [28], myoglobin [29], dsDNA [30,31], and drugs [32], as well as to reveal drug–DNA interactions [33]. PDMAEMA-based (co/ter)polymers were applied for the non-viral delivery of genetic material into cells [34,35]. PDMAEMA-based polymers were also reported as proper polymeric templates for the synthesis of metal NPs [36,37], wherein a strong stabilizing effect of PDMAEMA segments was reported [38].

In this work, we prepared metallopolymer hybrids consisting of AgNPs, which were templated on a poly(1,2-butadiene)_290_-*block*-poly(*N*,*N*-dimethylaminoethyl methacrylate)_240_ (PB-*b*-PDMAEMA) diblock copolymer (where the subscripts denote the number-average degrees of polymerization of the corresponding blocks). Apart from that, for the first time, we demonstrated local laser-induced enhancement of the SERS signal of 4-mercaptophenylboronic acid (4-MPBA) contacting with the obtained metallopolymer hybrid. Indeed, the possible local plasmonic heating of the AgNPs on PDMAEMA segments of the diblock copolymer under a laser irradiation might result in the heating of the polymer matrix in the laser spot and consequently initiate its phase transition (coil-to-globule transition of PDMAEMA segments followed by their subsequent aggregation). As assumed, the latter induces the approaching of AgNPs and thereby leads to the generation of “hot spots”. We also show herein that such a SERS triggering effect depends on the time of the laser exposure and on the concentration of 4-MPBA. Thus, we highlight a new approach to a SERS analysis that could imply potential applications in areas such as nanosensors for the detection and quantification of biologically relevant molecules.

## 2. Materials and Methods

### 2.1. Materials

4-MPBA, AgNO_3_, and NaBH_4_ were obtained from Sigma-Aldrich. The PB-*b*-PDMAEMA diblock copolymer was synthesized as described earlier [29]. All other chemicals were of analytical grade and used without further purification. All aqueous solutions were prepared using Milli-Q water (18.2 MΩ·cm) purified with a Milli-Q water purification system by Millipore.

### 2.2. Polymer-Templated Synthesis of AgNPs

For the synthesis of an AgNPs/PB-*b*-PDMAEMA sample, an aqueous solution of AgNO_3_ (24 µL, 0.1447 M) was first added to an alkaline solution of the PB-*b*-PDMAEMA diblock copolymer (3 mL, 0.023 M with respect to the monomer units of the polymer) at a Ag:N molar ratio of 1:20. The pH value of the mixture was carefully adjusted to pH 9 and the mixture was allowed to stay under stirring at room temperature for 30 min. Then, an aqueous solution of NaBH_4_ (130 µL, 2 g/L) was added into the mixture to provide a NaBH_4_:AgNO_3_ molar ratio of 2:1. Afterwards, the reaction mixture was stirred for 30 min at room temperature.

### 2.3. UV–Vis Spectroscopy

UV–Vis spectra were recorded using a Shimadzu UV-1800 double-beam spectrophotometer (Shimadzu, Kyoto, Japan) in quartz cuvettes with an optical path length of 10 mm. The spectra were acquired from 200 to 800 nm with a 1 nm resolution. Before acquisition, an AgNPs/PB-*b*-PDMAEMA sample was diluted 10 times with deionized water. The spectral data were treated with the ORIGIN software.

### 2.4. Transmission Electron Microscopy (TEM)

For TEM measurements, copper grids were rendered hydrophilic by Ar plasma cleaning for 2 min (Diener Electronics, Ebhausen, Germany). Ten microliters (10 µL) of either AgNPs/PB-*b*-PDMAEMA sample or control sample (AgNPs prepared in the absence of the PB-*b*-PDMAEMA diblock copolymer) were applied onto the grid and an excess of the sample was blotted with a filter paper. TEM images were acquired with a 200 kV FEI Tecnai G2 20 transmission electron microscope equipped with a 4 k × 4 k Eagle HS CCD and a 1 k × 1 k Olympus MegaView camera for overview images.

### 2.5. Turbidimetry

The transmittance of aqueous solutions of the PB-*b*-PDMAEMA diblock copolymer or aqueous dispersions of the AgNPs/PB-*b*-PDMAEMA metallopolymer hybrids with a concentration of 0.2 g/L (pH 9) was measured on a Shimadzu UV-1800 double-beam spectrophotometer (Shimadzu, Kyoto, Japan). The temperature-controlled sample holders were connected to a temperature controller allowing 6 simultaneous measurements. The samples were scanned at a fixed wavelength of 500 nm. Their light transmittance was recorded upon heating at a temperature variation rate of 0.4 °C/min. The inflection points on the transmittance–temperature curves, which correspond to the onset temperature of the transmittance decrease, are defined herein as the onset temperature of phase transition (T_PT_).

### 2.6. Raman Spectroscopy

An SERS signal was measured using an innoRam Raman spectrometer (BWTek, Plainsboro, NJ, USA) with a laser wavelength of 532 nm and power of 40 mW. The SERS-active analyte, that is, 4-MPBA, was used as a reporter as this compound can covalently bind to the Ag surface due to its mercapto group. Aqueous solutions of 4-MPBA were prepared at the concentrations of 3, 6, 15, and 30 μM and then mixed with an AgNPs/PB-*b*-PDMAEMA sample in a 1:1 ratio. Afterwards, the prepared mixtures were left for 30 min. Then, a 10 μL aliquot of each mixture was applied onto aluminum foil. The SERS spectra were recorded in between 5–8 cycles of continuous laser exposition (each of 1 min duration). The acquisition time was 10 s. The characteristic peak of 4-MPBA at 1070 cm^−1^ was used as an analytical signal for the evaluation of the SERS efficiency.

## 3. Results and Discussion

### 3.1. Physico-Chemical Characterization of the AgNPs Templated on the PB-b-PDMAEMA Micelles

In the present study, we used a stimuli-responsive (pH- and thermoresponsive) PB-*b*-PDMAEMA diblock copolymer, which comprises both a hydrophobic poly(1,2-butadiene) (PB) block with a low glass transition temperature (T_g_) of −15 °C and a hydrophilic (chargeable) poly(*N,N*-dimethylaminoethyl methacrylate) (PDMAEMA) block. The chemical structure of the PB-*b*-PDMAEMA diblock copolymer is shown in Figure S1A.

The stimuli-responsiveness of the PB-*b*-PDMAEMA diblock copolymer results from the presence of a PDMAEMA block. PDMAEMA is a weak polybase with each monomer unit containing a pendant tertiary amino group. A reversible protonation of such amino groups imparts pH sensitivity to the PB-*b*-PDMAEMA diblock copolymer. According to the published results [29,32], PDMAEMA undergoes a transition from the fully protonated (charged) state to the fully deprotonated (non-charged) state in a pH window of 4.0–8.5, and the pK_a_^app^ (at α = 0.5, where α is the degree of protonation of PDMAEMA) for the PB-*b*-PDMAEMA diblock copolymer was found to be 6.35. PDMAEMA is also related to thermosensitive polymers with cloud points of its aqueous solutions depending on the pH [23,24]. It is worth noting that the decreasing pH, which results in the charging of PDMAEMA due to the protonation of its monomer units, is accompanied by an increase in cloud points.

Being amphiphilic, the PB-*b*-PDMAEMA diblock copolymer self-assembles into micelles, which at the ambient temperature and neutral pH (α = 0.75) possess an overall average diameter of 106 nm. The micellar structure of the diblock copolymer was confirmed by means of cryogenic TEM (Figure S1B). Within a micelle, a hydrophobic PB core has a mean diameter of 38 nm while a hydrophilic PDMAEMA corona has a thickness of >34 nm [29]. The latter grants the formed PB-*b*-PDMAEMA micelles sufficient colloidal stability in aqueous media.

The in situ synthesis of AgNPs was performed by the pre-complexation of Ag^+^ ions with PDMAEMA blocks of the PB-*b*-PDMAEMA diblock copolymer, followed by their reduction to metallic Ag by sodium borohydride. The reduction was carried out at pH 9 by mixing an aqueous solution of AgNO_3_ with an aqueous solution of the PB-*b*-PDMAEMA micelles. The molar ratio of amino groups of PDMAEMA to AgNO_3_ (N:Ag) was kept at 20:1. According to our previously published results [25,29], all monomer units of PDMAEMA are uncharged (α = 0) at pH 9. Hence, the formation of an ion-coordination bond between an Ag^+^ ion and an uncharged N atom of a monomer unit of PDMAEMA was expected. The following reduction of Ag^+^ ions by a 2 molar excess of sodium borohydride results in the immediate appearance of a yellow-orange color, which indicates the formation of the AgNPs.

**Figure 1:**
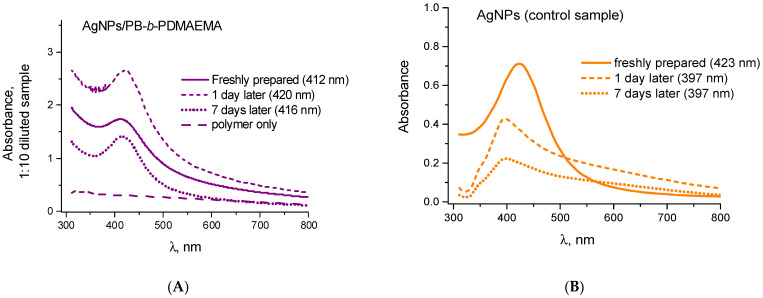
The time evolution of the UV–Vis spectra for the AgNPs prepared (**A**) in the presence and (**B**) in the absence of the PB-*b*-PDMAEMA micelles.

Figure 1A,B shows the UV–Vis spectra of the AgNPs formed in situ in the presence and in the absence of the PB-*b*-PDMAEMA diblock copolymer. As one can see, both samples, when freshly prepared, exhibit a characteristic surface plasmon resonance band at approximately 410–420 nm. While its position weakly depends on the presence or the absence of the copolymer, a considerable stabilization effect of the PB-*b*-PDMAEMA micelles is obvious. A spontaneous aggregation of non-stabilized AgNPs in the control sample results in a fast absorbance intensity decrease and broadening of the plasmonic peak soon after the preparation of AgNPs (Figure 1B). In solutions of the PB-*b*-PDMAEMA micelles, the plasmonic peak of the AgNPs keeps its position as well as its intensity (Figure 1A). One only notes certain (up and down) absorbance intensity variations for the metallopolymer hybrid system, which takes place during the first few days (Figure 1A). Afterwards, its absorbance demonstrates nearly no changes over at least 6 months (data not shown). The well-known strong stabilization effect of the PDMAEMA [38] is expected to impart such high colloidal stability to the AgNPs/PB-*b*-PDMAEMA metallopolymer hybrids. Apparently, the micellar structure of the PB-*b*-PDMAEMA diblock copolymer (Figure S1) could additionally contribute to the enhanced colloidal stability of the AgNPs/PB-*b*-PDMAEMA samples.

**Figure 2:**
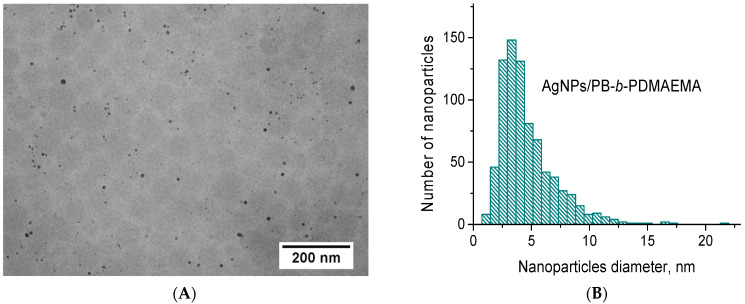
(**A**) The TEM image and (**B**) the corresponding size distributions of the AgNPs prepared in the presence of PB-*b*-PDMAEMA micelles.

The TEM images of the AgNPs that were in situ templated on the PDMAEMA blocks of the PB-*b*-PDMAEMA micelles are demonstrated in Figure 2A. Two types of objects are clearly visible in the obtained images. Numerous small darker spots are undoubtedly attributed to the AgNPs while light grey round-shape structures, which are significantly weaker in contrast, are thought to represent PB-*b*-PDMAEMA micelles in a dry state. More TEM images of the prepared AgNPs/PB-*b*-PDMAEMA metallopolymer hybrids are given in Figure S2.

Furthermore, the AgNPs formed in the presence of the PB-*b*-PDMAEMA micelles were mostly spherical-like ones, which were separated from each other (Figure 2A). Although one cannot definitely see how the AgNPs are distributed within the PB-*b*-PDMAEMA micelles, they are most likely located in the external (periphery) part of the micelles, that is, in the micellar corona built up by PDMAEMA blocks. Importantly, the TEM image of the control sample of AgNPs, which were synthesized in the absence of the PB-*b*-PDMAEMA diblock copolymer, considerably differs from that taken for the AgNPs/PB-*b*-PDMAEMA sample. Indeed, only aggregated AgNPs are observed in this case, as shown in Figure S3. These results further confirm a considerable stabilization of the AgNPs in these metallopolymer hybrids.

As follows from Figure 2B, the size distribution of the AgNPs formed in the presence of the PB-*b*-PDMAEMA micelles was found to be rather narrow. Their mean diameter was calculated as 4.6 ± 2.5 nm (n = 800). Assuming that all of them are isotropic and possess a crystalline structure with the closest-packed Ag atoms that form a *face-centered cubic lattice*, one can evaluate the mean number of Ag atoms per NP. Indeed, the volume of a lattice cell for a silver face-centered cubic lattice (a = b = c = 0.4086 nm) is V = 0.0682 nm^3^ with the number of Ag atoms per lattice cell of 4. Hence, one AgNP with a mean diameter of 4.6 ± 2.5 nm contains approximately 2990 atoms. If we assume that Ag atoms form less-packed clusters, then one can calculate the same for a *simple cubic lattice* (a = b = c = 0.288 nm, where 0.288 nm is a diameter of Ag atom). In this case, the volume of the lattice cell is V = 0.0239 nm^3^ with the number of Ag atoms per lattice cell of 1. Hence, one can evaluate approximately 2130 atoms per one AgNP with a mean diameter of 4.6 ± 2.5 nm. Thus, one can consider 2990 and 2130 values as an upper-bound estimate and a lower-bound estimate for the number of Ag atoms per one AgNP, respectively.

Keeping in mind that the molar ratio of N:Ag was set to 20:1 (see Section 2.2 for the conditions applied for the synthesis of the AgNPs), one can easily calculate the concentration of AgNPs in the AgNPs/PB-*b*-PDMAEMA system as 1.51 × 10^17^ particles/L (assuming a crystalline structure of the AgNPs) or 2.12 × 10^17^ particles/L (assuming a cluster structure of the AgNPs). Dividing these values by the concentration of the PB-*b*-PDMAEMA micelles (independently determined by means of nanoparticle tracking analysis as 3.7 × 10^16^ micelles/L [29]), one can further evaluate the mean number of the AgNPs per one micelle. Hence, one PB-*b*-PDMAEMA micelle apparently contains between 4 and 6 AgNPs. It is worth noting that these evaluations are in very good agreement with the analysis of the obtained TEM images (Figure 2A), wherein 5–6 AgNPs per one PB-*b*-PDMAEMA micelle were counted.

**Figure 3:**
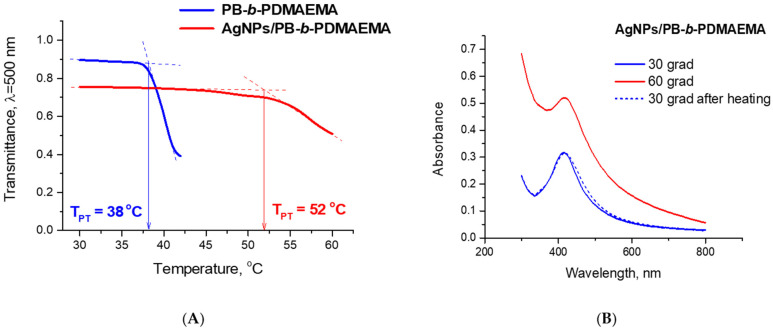
(**A**) Temperature-dependent changes in the transmittance of 0.2 g/L aqueous solutions of the PB-*b*-PDMAEMA micelles (blue line) and the AgNPs/PB-*b*-PDMAEMA hybrids (red line). (**B**) The UV–Vis spectra of 0.2 g/L aqueous solutions of the AgNPs/PB-*b*-PDMAEMA hybrids recorded at different temperatures. The pH values of all solutions were adjusted to pH 9 by 0.1 M NaOH.

A thermoresponsive behavior of the PB-*b*-PDMAEMA micelles and the prepared AgNPs/PB-*b*-PDMAEMA metallopolymer hybrids was revealed in our work by means of turbidimetry. The measurements were performed for 0.2 g/L solutions of each sample at a heating rate of 0.4 °C/min. A temperature-induced hydrophobization of the PDMAEMA blocks and the subsequent aggregation of the PB-*b*-PDMAEMA micelles resulted in a change in the transmittance of the samples (Figure 3A). The inflection points in the transmittance–temperature curves, which correspond to the onset temperature of the transmittance decrease, are defined herein as the onset temperature of the phase transition T_PT_. As shown in Figure 3A, the transmittance curves demonstrate temperature-induced phase transition for both the PB-*b*-PDMAEMA micelles and for the AgNPs/PB-*b*-PDMAEMA metallopolymer hybrids, although the intensity of the process and the temperature ranges are remarkably different. Indeed, the T_PT_ for the PB-*b*-PDMAEMA micelles was determined as 38 °C while the AgNPs/PB-*b*-PDMAEMA metallopolymer hybrids exhibit a higher T_PT_ of 52 °C (Figure 3A). Moreover, the phase transition in the latter case looks considerably less sharp and less pronounced. Apparently, the loading of PDMAEMA blocks with the AgNPs notably suppresses the temperature-induced phase transition. Furthermore, Figure 3B clearly demonstrates that the phase transition for the AgNPs/PB-*b*-PDMAEMA metallopolymer hybrids is completely reversible (at least, within the examined temperature cycle of 30 °C–60 °C–30 °C).

Thus, a successful synthesis of the AgNPs on a PB-*b*-PDMAEMA micellar template was confirmed by means of UV–Vis spectroscopy and TEM, wherein the shape and size of the AgNPs were determined. We also demonstrated a reversible character of the temperature-induced phase separation for the AgNPs/PB-*b*-PDMAEMA metallopolymer hybrids. These findings allow us to expect a compulsory aggregation of the AgNPs upon a laser beam exposure of the AgNPs/PB-*b*-PDMAEMA samples. Indeed, the plasmonic heating of AgNPs is thought to result in a temperature-induced collapse of PDMAEMA blocks (coil-to-globule transition) and their aggregation, thereby initiating the approaching of the AgNPs and appearance of “hot spots”. As consequence, the SERS effect is to be observed.

### 3.2. SERS for the AgNPs Templated on the PB-b-PDMAEMA Micelles

To reveal a potential of the AgNPs/PB-*b*-PDMAEMA metallopolymer hybrids to exhibit the SERS effect, the AgNPs were labeled with 4-MPBA. As it forms a strong Ag–S bond, this compound can covalently bind to AgNPs. The covalent bond formation also leads to the appearance of intense peaks in the SERS spectrum of 4-MPBA that renders it an outstanding reporter for SERS experiments with AgNPs. Moreover, boronic acid itself can covalently bind to polyols, including carbohydrates and glycated proteins, which can be advantageously exploited for the diagnosis of diabetes, thereby emphasizing the biochemical relevance of 4-MPBA [39].

The chemical structure and the exemplary SERS spectrum of 4-MPBA with characteristic vibrations is shown in Figure S4. The most intense peaks of 4-MPBA are at 414, 992, 1018, 1070, 1178, and 1570 cm^−1^. The peak at 1070 cm^−1^ is related to the in-plane benzene ring breathing mode coupled with the C–S stretching mode [40]. In further SERS experiments, the amplitude of this peak was used as an analytical signal.

**Figure 4:**
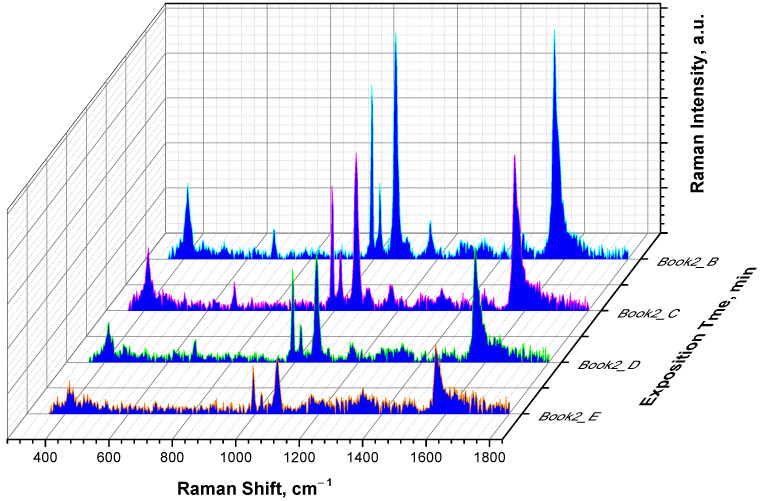
The evolution of the SERS spectra of the 10 μL drop of the AgNPs/PB-*b*-PDMAEMA hybrid with 30 μM of 4-MPBA at different exposure times: 0 min (orange line); 3 min (green line); 6 min (pink line); and 8 min (light blue line).

To demonstrate the SERS effect for the AgNPs/PB-*b*-PDMAEMA metallopolymer hybrids, a drop of the AgNPs/PB-*b*-PDMAEMA sample labeled with 4-MPBA was deposited on aluminum foil and then an initial SERS spectrum was acquired first. After that, the sample was continuously exposed to laser beam for 1 min. Then, a second SERS spectrum was acquired under the same conditions. This procedure was repeated eight times so that the total time of laser exposition was equal to 8 min. Figure 4 demonstrates that a continuous laser exposure leads to a notable increase in the intensity of the SERS spectra, the increase being proportional to the exposure time. This result is thought to be a consequence of the local “hot spot” generation through a laser-induced collapse of PDMAEMA blocks (coil-to-globule transition) and their aggregation, followed by AgNPs approaching.

**Figure 5:**
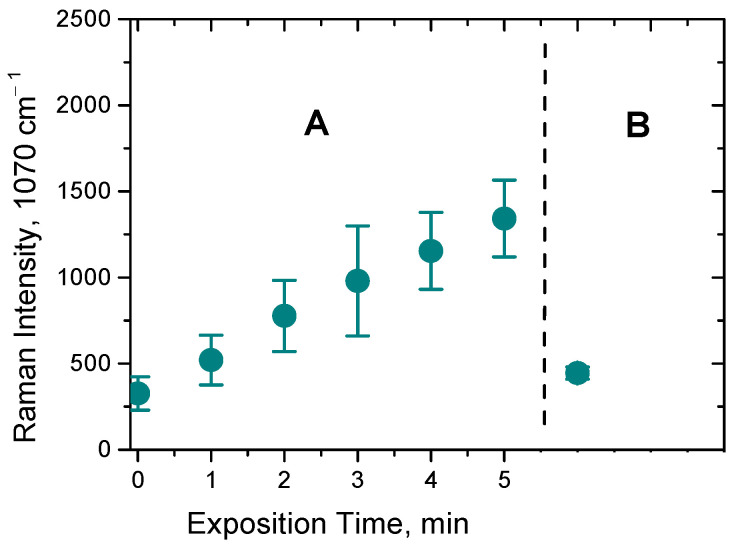
(**A**) The intensity increase in the 4-MPBA characteristic peak at 1070 cm^−1^ upon 5 cycles of a local laser exposure of a drop of the AgNPs/PB-*b*-PDMAEMA hybrid labeled with 4-MPBA; (**B**) The intensity of the 4-MPBA characteristic peak at 1070 cm^−1^ upon mixing a drop of the sample of the AgNPs/PB-*b*-PDMAEMA hybrid labeled with 4-MPBA. The data are presented as the mean ± SD for three independent experiments.

To prove whether the observed effect of the SERS intensity increase is due to a local laser exposure of the sample, a control experiment was performed, which started from recording the series of SERS spectra acquired in between five cycles of a 1 min laser exposure. Then, the laser was switched off, the drop of the sample was carefully mixed, and after that, one more SERS spectrum was recorded under the same conditions. As one can see from Figure 5, the intensity of the characteristic peak at 1070 cm^−1^ drops back to the initial value. Indeed, exchanging the exposed sample volume by a portion of the fresh sample via stirring leads to a decrease in the intensity of the SERS spectrum back to the start point, thus confirming that the local laser exposure plays a key role in the observed increase in the SERS signal.

**Figure 6:**
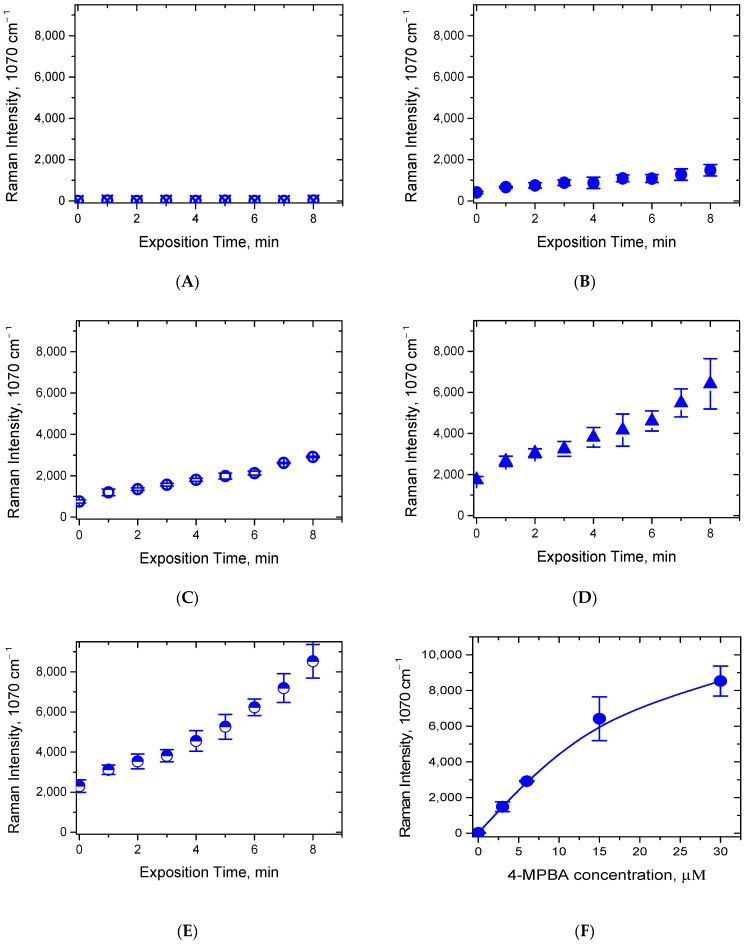
(**A**–**E**) The intensity increase in the 4-MPBA characteristic peak at 1070 cm^−1^ upon 8 cycles of local laser exposure of a drop of the AgNPs/PB-*b*-PDMAEMA hybrid labeled with 4-MPBA at different 4-MPBA concentrations (**A**) 0 μM (control with water), (**B**) 3 μM, (**C**) 6 μM, (**D**) 15 μM, and (**E**) 30 μM; (**F**) the dependence of the intensity of characteristic peak at 1070 cm^−1^ on 4-MPBA concentration plotted for the laser exposure time equal to 8 min. The data are presented as the mean ± SD for three independent experiments.

Furthermore, we recorded the SERS spectra in the same manner at different 4-MPBA concentrations and compared the intensities of the characteristic peak at 1070 cm^−1^ at different times of laser exposure. As one can see from Figure 6B–E, eight 1 min cycles of the continuous laser exposure lead to a notable increase in the start point, the endpoint, and the signal growth rate of the SERS signal intensity, the increase being proportional to the time of the laser exposure and to the concentration of 4-MPBA. A close to linear signal growth with the laser exposure is evident, although some deviations from linearity are found at the high concentrations of 4-MPBA. One can also note a concentration dependence of the initial SERS signal in the start point. This might be a consequence of a certain initial aggregation of the AgNPs, which could be induced by 4-MPBA upon its mixing with the AgNPs/PB-*b*-PDMAEMA sample. A control sample, which was measured in the absence of 4-MPBA, demonstrates no SERS signal (Figure 6A). One more control sample measured at 30 μM of 4-MPBA in the absence of the AgNPs/PB-*b*-PDMAEMA metallopolymer hybrids shows no SERS signal either (data not shown). Here, the time of the laser exposure was limited to 8 min to avoid the significant evaporation of the sample. The evaporation of a 10 μL drop was followed in a separate experiment and was found to be not exceeding 20% of the drop volume.

The endpoint intensities of the characteristic peak at 1070 cm^−1^ were plotted against the concentration of 4-MPBA (Figure 6F). By this experiment, we demonstrated a potential benefit of such local triggering effects for the AgNPs/PB-*b*-PDMAEMA metallopolymer hybrids in the context of the SERS sensing applications. We think that the new approach considered herein could significantly broaden the applied range of SERS sensing, which could have possible applications in areas such as nanosensors for biologically relevant molecules.

### 3.3. Theoretical Simulation of Sample Heating by a Laser Beam in SERS Experiments

To confirm that a laser exposure of the AgNPs/PB-*b*-PDMAEMA sample in the SERS experiments leads to heating that is sufficient for the temperature-induced collapse and aggregation of PDMAEMA blocks, a theoretical consideration of sample heating by a laser beam was carried out. The temperature distribution in a sample drop was simulated, based on the volume-specific heat source distribution resulting from the absorption of the laser light by AgNPs. The absorbance spectra (Figure 1A) of the diblock copolymer solutions with and without the AgNPs were used to determine the light absorption per unit volume. It was assumed that the extinction of a solution without the AgNPs resulted from the light scattering by PB-*b*-PDMAEMA micelles. Then, the difference between the extinctions of solutions with and without the AgNPs is expected to be exclusively due to light absorption in the AgNPs. Here, we neglected the own light scattering of the AgNPs since for the AgNPs with a size distribution as in our samples (Figure 2B), the average absorption cross-section is approximately an order of magnitude larger than the average scattering cross-section at λ = 532 nm. To determine the volume-specific heat source distribution, the absorption coefficient of the AgNPs/PB-*b*-PDMAEMA sample (determined from the difference between the extinction spectra of the samples with and without the AgNPs) and the exponential attenuation of the laser intensity (determined from the entire extinction of the sample with the AgNPs) were taken into account.

**Figure 7:**
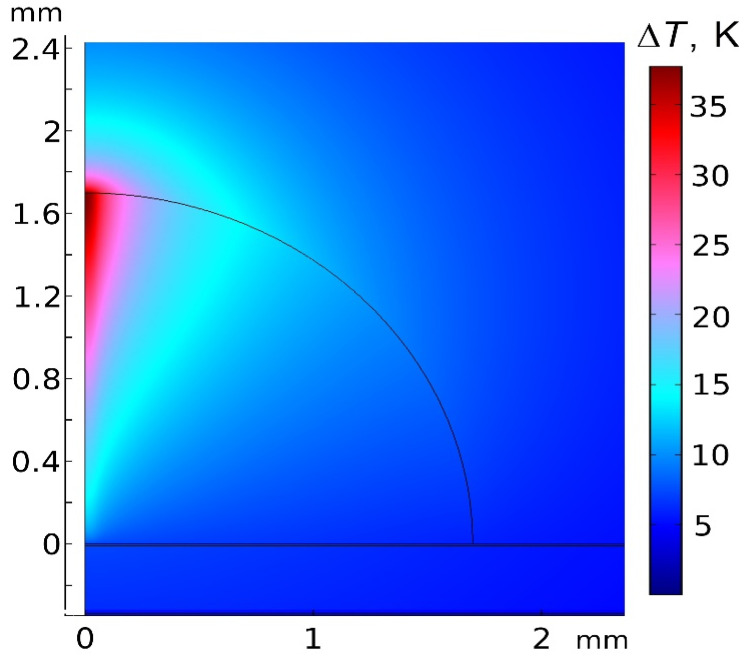
The simulated temperature increase distribution (with respect to the room temperature) resulting from laser heating in a sample drop (by a volume of 10 μL) on a foil and in the surrounding air (in the axisymmetric geometry). The horizontal direction corresponds to the radial coordinate (the symmetry axis is perpendicular to the foil plane and coincides with the laser beam axis).

Figure 7 shows the temperature increase distribution (relative to the room temperature) in a sample drop lying on an aluminum foil and in the surrounding media in the steady state. The maximum temperature increase in the sample was assessed as 37.7 K with respect to room temperature, which is sufficient for PDMAEMA blocks to collapse and aggregate as experimentally shown (Figure 3A). We assume that the SERS signal increase could be explained by the gradual temperature-induced transformation of the polymer in the steady temperature distribution (according to [19,20], the temperature-induced collapse time of thermoresponsive polymers might take approximately hundreds of seconds).

## 4. Conclusions

In summary, we synthesized the AgNPs templated on the thermoresponsive PB-*b*-PDMAEMA micelles and showed a temperature-induced reversible phase transition of the prepared AgNPs/PB-*b*-PDMAEMA metallopolymer hybrids. With a SERS reporting probe (4-MPBA), we further revealed a laser-induced enhancement of a SERS signal observed under a constant local laser irradiation. The triggered SERS effect depends on the time of the laser exposure and on the concentration of 4-MPBA. The local heating of the AgNPs/PB-*b*-PDMAEMA sample in a laser spot is in good agreement with a performed theoretical consideration pointing to a temperature increase up to approximately 38 °C. A plasmonic heating of the AgNPs during the local laser exposure of the AgNPs/PB-*b*-PDMAEMA sample is thought to be responsible for the triggered SERS effect. Indeed, the plasmonic heating initiates a collapse of the PDMAEMA blocks and their gradual aggregation, which leads to the approaching of the AgNPs and generation of “hot spots”. However, an optical trapping effect (optical tweezers) that might appear as a result of a decreasing distance among the AgNPs as reported in [21] could also be a reasonable explanation.

We think that the smart stimuli-responsive metallopolymer hybrid materials reported herein could have promising applications in areas such as SERS nanosensors for the detection and quantification of biologically relevant molecules. At the same time, further optimization of the technique and the experimental conditions appears to be necessary to improve the approach and to increase the sensitivity, both of which will be subjects of future work.

## Data Availability

Research data are not shared.

## Supplementary Materials

The following supporting information can be downloaded at: https://www.mdpi.com/article/10.3390/bios12080628/s1, Figure S1: The chemical structure of the PB-b-PDMAEMA (A) and the cryo-TEM micrograph of the PB-b-PDMAEMA micelles (B) in 50 mM sodium phosphate (c = 2.5 g/L) at pH 7.0. Figure S2: The TEM images of the AgNPs/PB-b-PDMAEMA hybrids taken from different places of the sample at different magnification. Figure S3: The TEM image of the control AgNPs sample that was prepared under the same conditions in the absence of the PB-b-PDMAEMA micelles. Figure S4: The typical SERS spectrum of 4-MPBA with characteristic vibrations.
