# Supplementary material for: In Situ SERS Sensing by a Laser-Induced Aggregation of Silver Nanoparticles Templated on a Thermoresponsive Polymer
Source: Biosensors (Basel). 2022 Aug 11;12(8):628. doi: 10.3390/bios12080628 (PMC9405980; doi:10.3390/bios12080628)
Supplement: Supplementary file 1 [file biosensors-12-00628-s001.zip › biosensors-1798112-supplementary.pdf]

biosensors

Supporting Information

In situ SERS sensing by a laser-induced aggregation of silver nanoparticles templated on a thermoresponsive polymer

Larisa V. Sigolaeva [1*], Natalia L. Nechaeva [2], Anton I. Ignatov [3,4], Lyubov Y. Filatova [1], Timur Z. Sharifullin [1], Jonas Eichhorn [5], Felix H. Schacher [5,6,7], Dmitry V. Pergushov [1,8], Alexander M. Merzlikin [9] and Ilya N. Kurochkin [1,2*]

[1] M.V. Lomonosov Moscow State University, Department of Chemistry, 119991 Moscow, Russia
[2] N.M. Emanuel Institute of Biochemical Physics of Russian Academy of Sciences, 119334 Moscow, Russia
[3] All-Russia Research Institute of Automatics, 127055 Moscow, Russia
[4] National Research Moscow State University of Civil Engineering, 129337 Moscow, Russia
[5] Institute of Organic Chemistry and Macromolecular Chemistry (IOMC), Friedrich-Schiller-University Jena, D-07743 Jena, Germany
[6] Jena Center for Soft Matter (JCSM), Friedrich-Schiller-University Jena, D-07743 Jena, Germany
[7] Center for Energy and Environmental Chemistry (CEEC), Friedrich-Schiller-University Jena, D-07743 Jena, Germany
[8] N.N. Semenov Federal Research Center of Chemical Physics of Russian Academy of Sciences, 119991 Moscow, Russia
[9] Institute of Theoretical and Applied Electromagnetics of Russian Academy of Sciences, 125412 Moscow, Russia
* Correspondence: lsigolaeva@belozersky.msu.ru (L.V.S.); Tel.: +7 495 939 40 42; ikur@sky.chph.ras.ru (I.N.K.) Tel.: +7 495 939 43 91

-

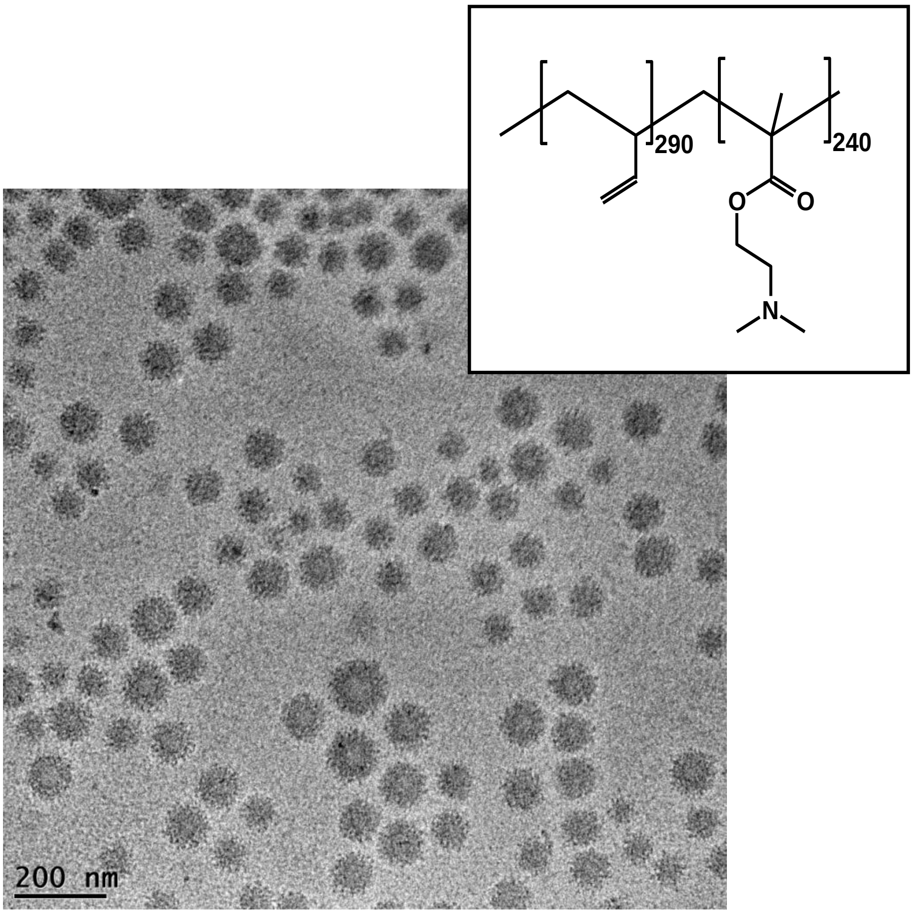

Figure S1. The chemical structure of the PB-*b*-PDMAEMA (A) and the cryo-TEM micrograph of the PB-*b*-PDMAEMA micelles in 50 mM sodium phosphate (c = 2.5 g/L) at pH 7.0 (B).

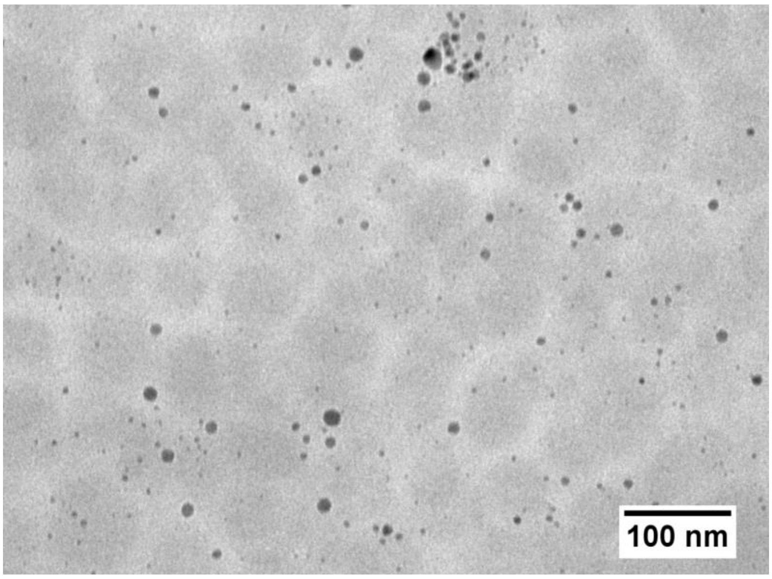

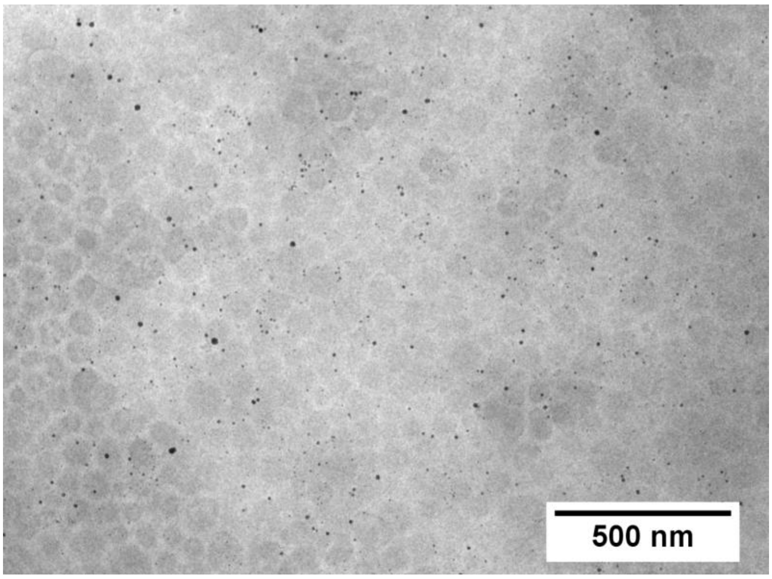

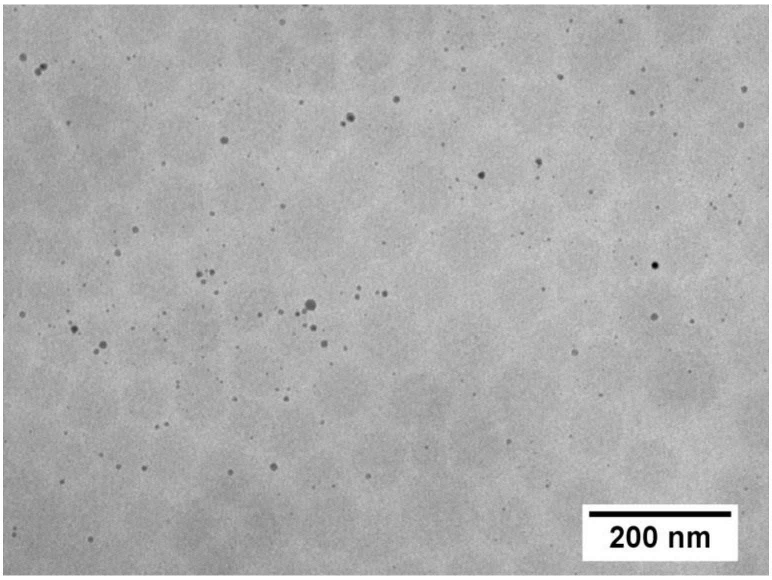

Figure S2. The TEM images of the AgNPs/PB-*b*-PDMAEMA hybrids taken from different places of the sample at different magnification.

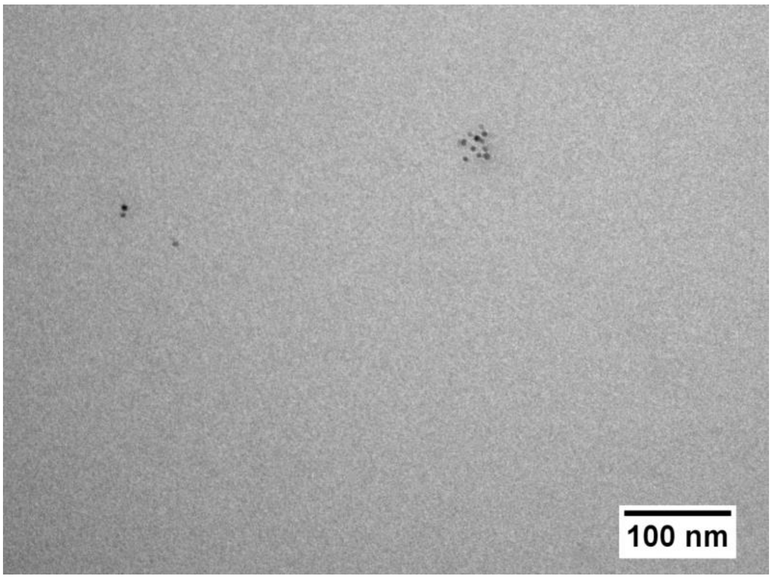

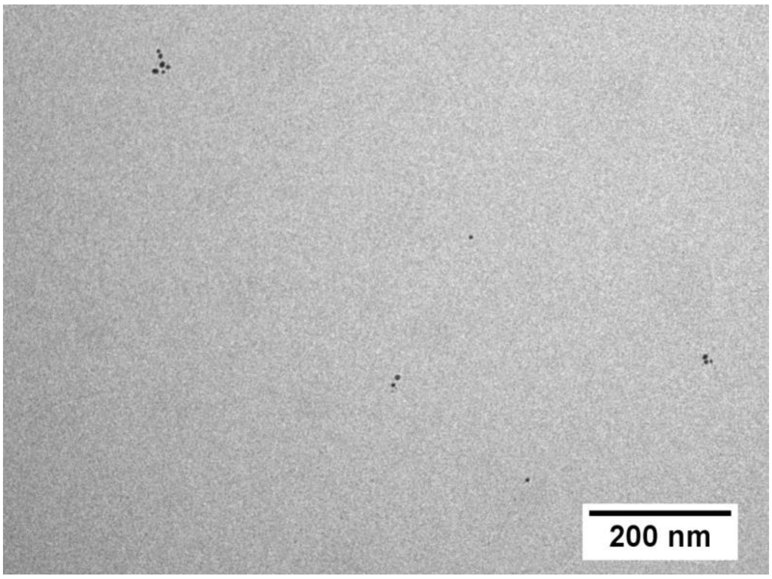

Figure S3. The TEM images of the control AgNPs sample that was synthesized under the same conditions in the absence of the PB-*b*-PDMAEMA micelles.

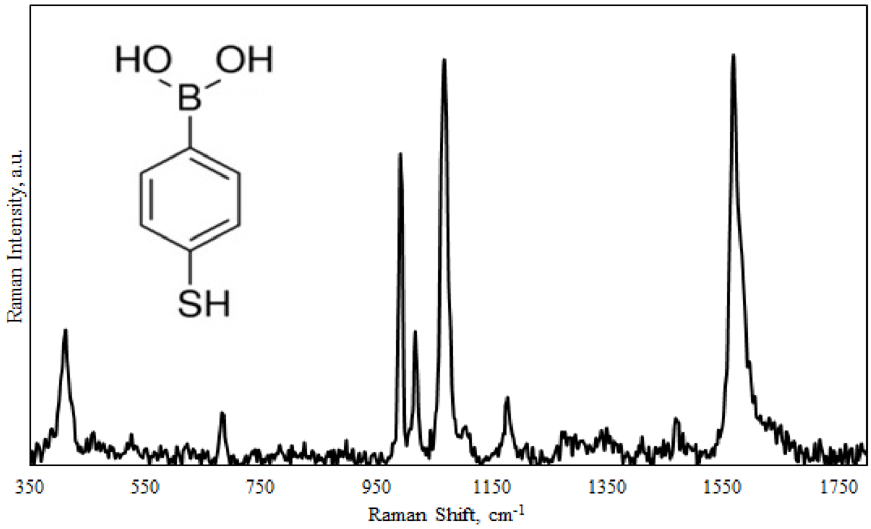

Figure S4. The SERS spectrum of 4-MPBA with characteristic vibrations. Inset: the chemical structure of 4-MPBA. The peak at 1070 cm^{-1}, which is related to the in-plane benzene ring breathing mode coupled with the C-S stretching mode, was used as an analytical signal for the SERS experiments. Conditions: an aqueous solution of 4-MPBA with the concentration of 30 μM was mixed with the sample of the AgNPs/PB-*b*-PDMAEMA hydrid in a 1:1 ratio, after 30 minutes a 10 μl aliquot of the mixture was applied onto aluminium foil and the SERS spectrum was acquired.
